# Translational deep learning models for risk stratification to predict prognosis and immunotherapy response in gastric cancer using digital pathology

**DOI:** 10.1186/s12967-025-07416-z

**Published:** 2025-12-24

**Authors:** Mai Hanh Nguyen, Huy-Hoang Do-Huu, Phuc-Tan Nguyen, Ngoc Dung Tran, Nguyen Thuy Linh, Hieu Le, Nguyen Quoc Khanh Le

**Affiliations:** 1 International Ph.D. Program in Cell Therapy and Regenerative Medicine, College of Medicine, Taipei Medical University, Taipei, 110 Taiwan; 2 Department of Pathology and Forensic Medicine, 103 Military Hospital, Hanoi, Vietnam; 3 AIBioMed Research Group, Taipei Medical University, Taipei, 110 Taiwan; 4 University of Science, VNU-HCM, Ho Chi Minh City, Vietnam; 5 Vietnam National University, Ho Chi Minh City, Vietnam; 6 Department of Computer Science, University of North Carolina at Charlotte, Charlotte, NC 28223 USA; 7 In-Service Master Program in Artificial Intelligence in Medicine, College of Medicine, Taipei Medical University, Taipei, Taiwan; 8 Translational Imaging Research Center, Taipei Medical University Hospital, Taipei, 110 Taiwan

**Keywords:** Gastric cancer, Whole-slide image, Deep learning, Immunotherapeutic response, Prognoses

## Abstract

**Background:**

Gastric cancer (GC) is one of the leading causes of cancer-related deaths globally, with a 5-year survival rate of less than 40%. While immune checkpoint inhibitors have provided promising therapeutic options for advanced GC, only a small proportion of patients benefit. In this study, we developed a deep learning model using whole-slide images to predict prognoses and sensitivity to immune checkpoint inhibitors in GC patients by predicting a novel marker.

**Methods:**

Formalin-fixed, paraffin-embedded whole-slide images from 292 patients in the Cancer Genome Atlas-Stomach Adenocarcinoma cohort were analyzed. Tumor regions were identified using a ResNet50-based tumor detection model and validated in HiESD dataset. Tiles classified as malignant were extracted for subsequent analysis. Risk score prediction models were developed using convolutional neural networks, clustering-constrained attention multiple-instance learning (CLAM), and dual-stream multiple-instance learning (DSMIL). Attention heatmap visualization was used to interpret tumor microenvironment (TME) features and a multi-model classification framework utilizing a support vector machine (SVM) was developed to assess the impact of clinical variables in model performances. The results were evaluated using the area under the receiver operating characteristic curve (AUROC), accuracy, sensitivity, specificity, and F1 score.

**Results:**

The tumor detection model achieved an AUROC of 0.99 on the training set, 0.92 on the test set, and 0.87 on external test set. Among risk score prediction models, DSMIL demonstrated the highest performance, with an AUROC of 0.73 and accuracy of 0.73 on the training set and AUROC of 0.70 and accuracy of 0.68 on the internal test set. High-risk patients exhibited worse survival outcomes and lower immunotherapy response rates compared with low-risk patients. Feature attribution analysis using attention heatmaps confirmed that the model prioritized TME components, specifically regions with dense lymphocytic infiltration. The muti-modal analysis showed that the image features alone were superior to the model combining image features with clinicopathological data.

**Conclusion:**

Deep learning models leveraging whole-slide images show potential in predicting prognoses and immunotherapy responses in GC. By integrating tumor-specific and tumor microenvironmental features, this approach offers a scalable, objective tool for personalized treatment planning, improving precision oncology strategies.

**Supplementary Information:**

The online version contains supplementary material available at 10.1186/s12967-025-07416-z.

## Background

Gastric cancer (GC) remains one of the most prevalent and lethal malignancies worldwide, with survival rates significantly varying based on the disease stage at diagnosis and available treatment options [1]. Overall 5-year survival rates for GC are still quite low, at approximately 29.9% for men and 39.2% for women [2]. In recent years, the emergence of immunotherapy, particularly immune checkpoint inhibitors (ICIs), introduced new therapeutic opportunities for patients with advanced GC [3–5]. Although this treatment method was proven to be very effective, not all GC patients benefit from ICIs. KEYNOTE-012, a study on the safety of pembrolizumab in treating advanced GC, found that 53% of patients had various degrees of tumor reduction [6]. Another study named KEYNOTE-059, which focused on the clinical efficacy of pembrolizumab in patients with disease progression, found that the objective response rate was 11.6% [7]. For patients with advanced GC squamous cell carcinoma or adenocarcinoma that is refractory to chemotherapy, ICIs demonstrated durable responses, with an observed median duration of response of 18.0 months [8]. However, most patients (~ 85%) exhibit primary resistance and do not benefit from ICI monotherapy. Predicting which patients will benefit from immunotherapy and assessing their prognosis remain critical challenges due to tumor heterogeneity, the immune system’s interaction with the tumor, genetics, and epigenetic alterations [9, 10]. Traditional clinical markers, while useful, often fall short of providing a comprehensive understanding of the tumor biology and its interaction with the immune system. Thus, effective predictive tools and biomarkers are needed in clinical practice to characterize and select advanced GC patients who are likely to respond to ICIs, aiming to improve the efficiency of immunotherapy in clinical applications.

Whole-slide images (WSIs), a high-resolution digital format of histopathological slides, have emerged as an invaluable resource for detailed tumor analyses [11]. This format has revolutionized the way histopathological slides are analyzed, providing a powerful tool for enhancing diagnostic accuracy and prognostic predictions. Traditionally, examining tissue samples under a microscope has been a cornerstone of cancer diagnosis and classification. However, this manual approach is subject to interobserver variability, limiting the reproducibility and consistency of results. With the advent of digital pathology, WSIs can be digitized and analyzed computationally, offering a more objective and scalable solution. With the advent of artificial intelligence (AI) and deep learning, novel approaches to analyzing medical images have shown promise in transforming the landscape of cancer prognosis and treatment.

Recent research identified some biomarkers and indicators that improve the prediction of immunotherapy responses, complementing traditional markers like programmed death-ligand 1 (PD-L1), the tumor mutational burden (TMB), and microsatellite instability (MSI). In addition, there are some AI applications for predicting immunotherapy for GC from WSIs such as predicting molecular features for immunotherapy-sensitive subtypes [12], or predicting patient sensitivity to first-line combined PD-1 inhibitor chemotherapy based on pretreatment biopsy samples [13]. Besides the effect on the immune system, hypoxia is also a crucial tumor microenvironment (TME) factor that can affect GC patient progression and response to this treatment [14–17]. Our previous study successfully identified a novel biomarker that combined both hypoxia and immune status and then calculated a risk score that can predict immunotherapy responses for GC patients with high reliability. GC patients with high-risk scores had shorter overall survival times and poorer responses to immunotherapy [18]. When developing models from pathological images, some studies utilized features extracted specifically from the tumor area [12, 19, 20], while others derived features from the entire tissue region [21, 22]. Thus, we conducted this study to achieve two aims: (1) to establish deep learning (DL) models to predict patients’ sensitivity to ICIs with high performance; and (2) to investigate whether a model’s performance is influenced by features extracted from the tumor area versus those from the TME on WSIs.

## Materials and methods

### Patient selection

Our study consisted of hematoxylin and eosin (H&E)-stained formalin-fixed, paraffin-embedded (FFPE) digital slides from The Cancer Genome Atlas (TCGA)-Stomach Adenocarcinoma (STAD). We collected high-resolution pathological images from 292 individuals with clinical information. The diagnostic slides were downloaded in SVS format. Any slides with tissue folds, blurred artifacts, or pen marks were excluded. According to the WSIs from TCGA-STAD, the risk score for each patient from our previous study was selected for further analysis. Additionally, for tumor detection task, we performed external validation using the multi-center HiESD dataset from the main hospital at the first affiliated hospital of Xi’an Jiaotong University [23].

### Pre-processing

All slide images were downloaded from TCGA database at 20× magnification. First, each input image was split into fixed-size tiles of 224 × 224 pixels (0.46 μm/pixel). Then tissue segmentation was performed using Otsu’s method, which automatically determines an optimal pixel intensity threshold based on the image histogram. A fixed intensity threshold of 200 was then applied to select tissue patches. Only patches containing a sufficient number of pixels with intensities above this threshold were considered valid tissue regions. This procedure allowed for effective exclusion of non-tissue areas while preserving meaningful histopathological structures for subsequent analysis.

### Tumor detection

In this study, we assessed the performance of various AI algorithms. In the first step, we hypothesized that the key information for predicting the risk score status is primarily located within the tumor tissues, specifically carcinomatous cells, excluding the surrounding stroma. To test this hypothesis, we trained a tumor-detection model to identify and classify tumor tiles.

Considering the accuracy and efficiency of the tumor-detection model for annotation, we utilized two datasets. For the first dataset, we randomly selected 20 WSIs. To establish the second dataset, we collected an additional 20 WSIs. The purpose of this setup was to evaluate the number of slides required for annotation to achieve effective model performance. From each WSI, 400 tiles were randomly extracted, resulting in a total of 8000 tiles for dataset 1 and 16,000 tiles for dataset 2. Additionally, we designated an internal test set for each dataset by randomly selecting five WSIs, corresponding to 2000 tiles per test set. We also reserved an external test set consisting of five slides from HiESD database, which corresponded to a total of 2,000 tiles, with 400 tiles sampled from each slide for external testing. Two expert pathologists, with 7 and 15 years of experience, independently labeled each tile as normal or malignant. In cases of disagreement, the final diagnosis was determined by the opinion of a third expert pathologist. Then we used the ResNet50 model pretrained on ImageNet to constitute the backbone of feature extraction. The output of ResNet50 was the probability of these tiles being malignant. These tumor tiles were used in the second step classifier.

### Risk score classification models

We employed three distinct approaches to develop a model to predict a risk score for GC patients based on WSIs. To construct and evaluate the models, we partitioned the dataset into training and internal test subsets, using an 85%/15% split. This ensured that the model was trained on a sufficient amount of data while keeping a separate set to test its ability to generalize. Within the training subset, we implemented a 5-fold cross-validation strategy, which is a reliable technique for preventing overfitting and assessing a model’s performance across various data subsets. For this method, the training set was divided into five equal segments, with four parts used for training and one part reserved for validation. This process allowed us to fine-tune the model’s hyperparameters, including the learning rate, optimizer, regularization, and batch size. Throughout the entire training procedure, the validation set remained an essential component of the training process.

### Base classification model

Convolutional neural networks (CNNs) are among the most effective DL architectures for handling image data due to their ability to extract spatial hierarchies of features, from low-level edges to high-level patterns. In this study, we employed a base CNN model tailored for our classification task by customizing fully connected (FC) layers to include four hidden layers. The convolutional layers served as feature extractors, while pooling layers reduced dimensionality, and Rectified Linear Unit (ReLU) activation introduced non-linearity. Each hidden layer was followed by a dropout layer to mitigate overfitting, ensuring generalizability during training.

### Clustering-constrained attention multiple-instance learning (CLAM) model [24]

In addition to the base CNN model, we employed a CLAM model, which was specifically designed for weakly supervised learning on WSIs. The CLAM model addressed challenges such as the gigapixel size of WSIs and the heterogeneity of tumor regions by leveraging attention mechanisms that focused on the most informative regions of the WSI. After hyperparameter tuning, the model was trained with 50 epochs and a batch size of 1.

### Dual-stream multiple-instance learning (DSMIL) model [25]

The third approach utilized a DSMIL model, designed to directly train on entire WSIs and address challenges of gigapixel-scale images. Unlike patch-based methods, the DSMIL model holistically processes WSIs, leveraging a dual-stream architecture that combines instance-level and bag-level information. The instance-level stream extracts fine-grained features from individual patches, capturing local tissue characteristics, while the bag-level stream aggregates these features to represent global contextual information. This dual-stream mechanism integrates localized and global patterns, enhancing predictive accuracy and enabling comprehensive analysis of complex WSI data. After hyperparameter tuning, the model was trained with 50 epochs, a learning rate of 0.01, a patch dropout rate of 0.1, a bag classifier dropout rate of 0.1, and weight decay of 0.001.

### Integration of deep features and clinicopathological data

We developed a multi-modal classification framework to investigate whether the integration of clinical factors could enhance the predictive performance achieved by image morphology alone. The clinical features integrated included survival status, age, stage, pathological subtype, T stage, N stage, and sex. For each sample, the pre-computed bag-level slide features (a 512-dimensional embedding extracted by the DSMIL model) were concatenated with the preprocessed clinical data. The preprocessing pipeline involved Z-score normalization for numerical features and one-hot encoding for categorical features. The core classification model implemented was a Support Vector Machine (SVM) using the scikit-learn’s SVC class, configured with class weighting set to ‘balanced’ and probabilistic outputs enabled. Finally, we performed a systematic grid search over key hyperparameters, specifically the regularization parameter C ∈ {0.001,0.01,0.1,1,10,100}, the kernel coefficient γ ∈ {0.01,0.1,1,10,100}, and the kernel type ∈ {‘sigmoid’, ‘rbf’} to identify the optimal configuration for this multi-modal approach. For each parameter combination, the model was trained on the training dataset and evaluated on the internal validation dataset.

### Statistical analysis

**Fig. 1:**
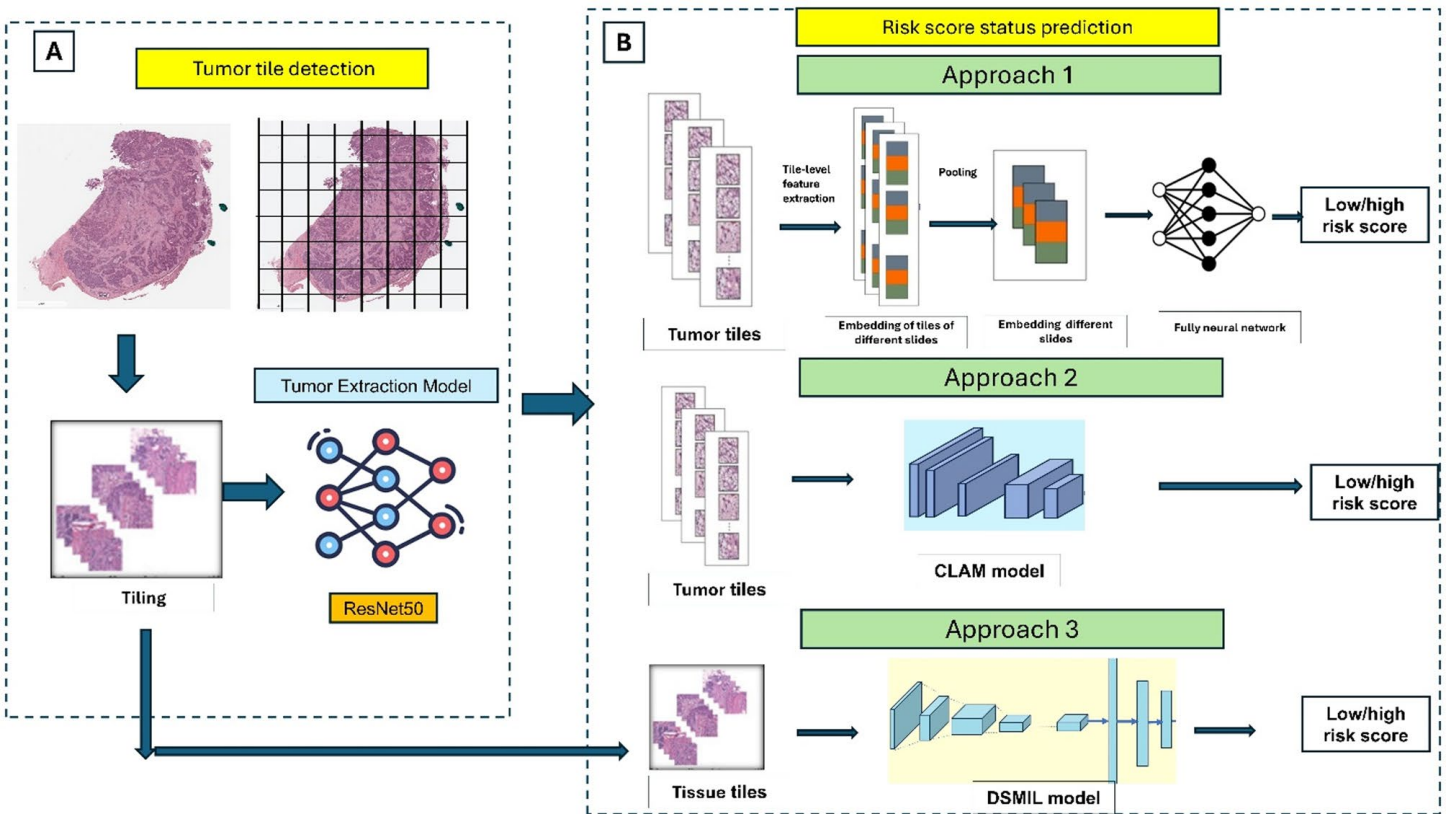
Overview of **t**he pipeline for the risk score prediction model. **A.** Tumor tile detection model: whole-slide images (WSIs) were processed by tiling the tissue region, removing the background, and putting the tiles into ResNet50 to extract tumor tiles. **B**. Risk score prediction models. Convolutional neural network (CNN) and clustering-constrained attention multiple-instance learning (CLAM) models used features from tumor tiles to make predictions, while dual-stream multiple-instance learning (DSMIL) utilized tissue tiles

We applied *t*-tests and Chi-squared tests to assess differences between patients in the training set and internal test set. To measure the binary classification performance of tile-based models on the test data, we utilized several key evaluation methods of (1) a receiver operating characteristic (ROC) curve; (2) area under the ROC curve (AUROC); (3) F1 score; (4) accuracy; and (5) confusion matrix to compute important performance metrics such as accuracy, specificity, sensitivity, negative predictive value (NPV), and positive predictive value (PPV) to assess the model’s overall performance. To assess the statistically significant difference in the predictive performance between CLAM And DSMIL, we used DeLong’s test. The attention heatmap visualization was employed as a qualitative analysis tool to generate spatial maps across the WSIs and mechanistically interpret the TME components that the model prioritized for risk score prediction. All analyses were conducted using Python 3.7 with the Pytorch framework. Computational tasks were carried out on Google Colab Pro+ (Google, Mountain View, CA, USA), utilizing an NVIDIA Tesla P100 GPU with 25 GB of RAM. Our full pipeline of prediction model is shown in Fig. 1.

## Results

### Patient characteristics

We collected data on 292 patients with clinical information, with 248 patients in the training set and 44 patients in the internal test set, among whom 134 were high-risk patients and 158 were low-risk patients. Mean ages were comparable between the two cohorts, at 65.13 ± 10.9 years in the training set and 65.3 ± 9.95 years in the internal test set (*p* > 0.05). Moreover, there was a similar ratio of genders of the patients, the clinical status, and the laterality of the images. Results are demonstrated in Table 1.

**Table 1:** Patient characteristics in the training set and internal test set

Characteristic	Training set	Internal test set	*p* value
Mean age (years)	65.13 ± 10.9	65.3 ± 9.95	0.922
Sex			
Male	163 (65.7%)	28 (63.6%)	0.923
Female	85 (34.3%)	16 (36.4%)	
T stage			
T1	1 (0.4%)	2 (4.5%)	0.086
T2	40 (16.1%)	8 (18.2%)	
T3	115 (46.4%)	20 (45.5%)	
T4	92 (37.1%)	14 (31.8%)	
N stage			
N0	75 (30.2%)	12 (27.3%)	0.926
N1	70 (28.2%)	12 (27.3%)	
N2	46 (18.6%)	10 (22.7%)	
N3	57 (23%)	10 (22.7%)	
Survival status			
Alive	150 (60.5%)	29 (65.9%)	0.608
Dead	98 (39.5%)	15 (34.1%)	
Stage			
I	29 (11.7%)	7 (15.9%)	0.679
II	70 (28.2%)	9 (20.5%)	
III	125 (50.4%)	24 (54.5%)	
IV	24 (9.7%)	4 (9.1%)	
Risk score			
High risk	115 (46.4%)	19 (43.2%)	0.820
Low risk	133 (53.6%)	25 (56.8%)	

### Extraction of tumor tiles

**Fig. 2:**
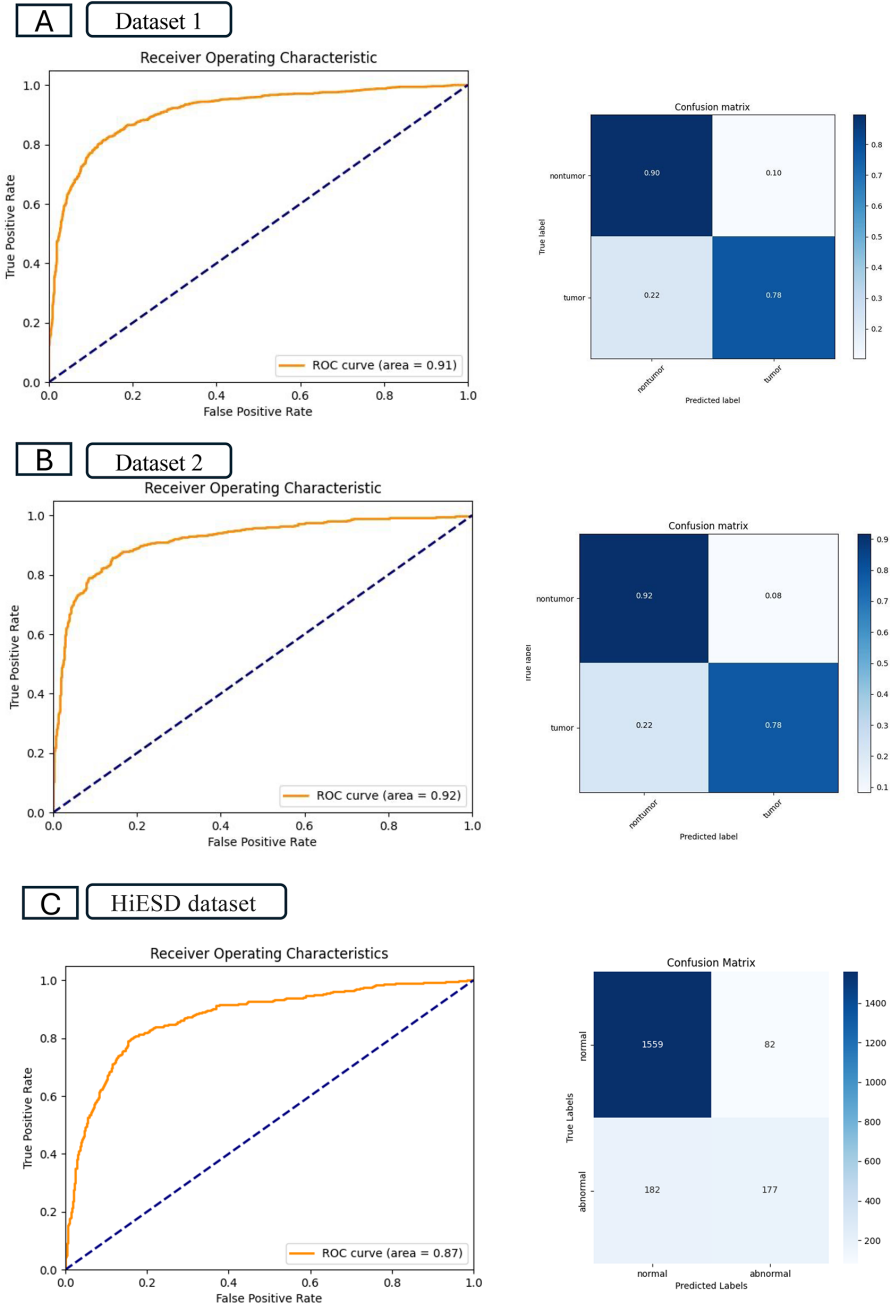
Performance of the model in tumor extraction. **A** The receiver operating characteristics curves, area under the curve, and confusion matrix of dataset (1) **B** The receiver operating characteristics curves, area under the curve, and confusion matrix of dataset (2) **C**. The receiver operating characteristics curves, area under the curve, and confusion matrix of external test set

**Fig. 3:**
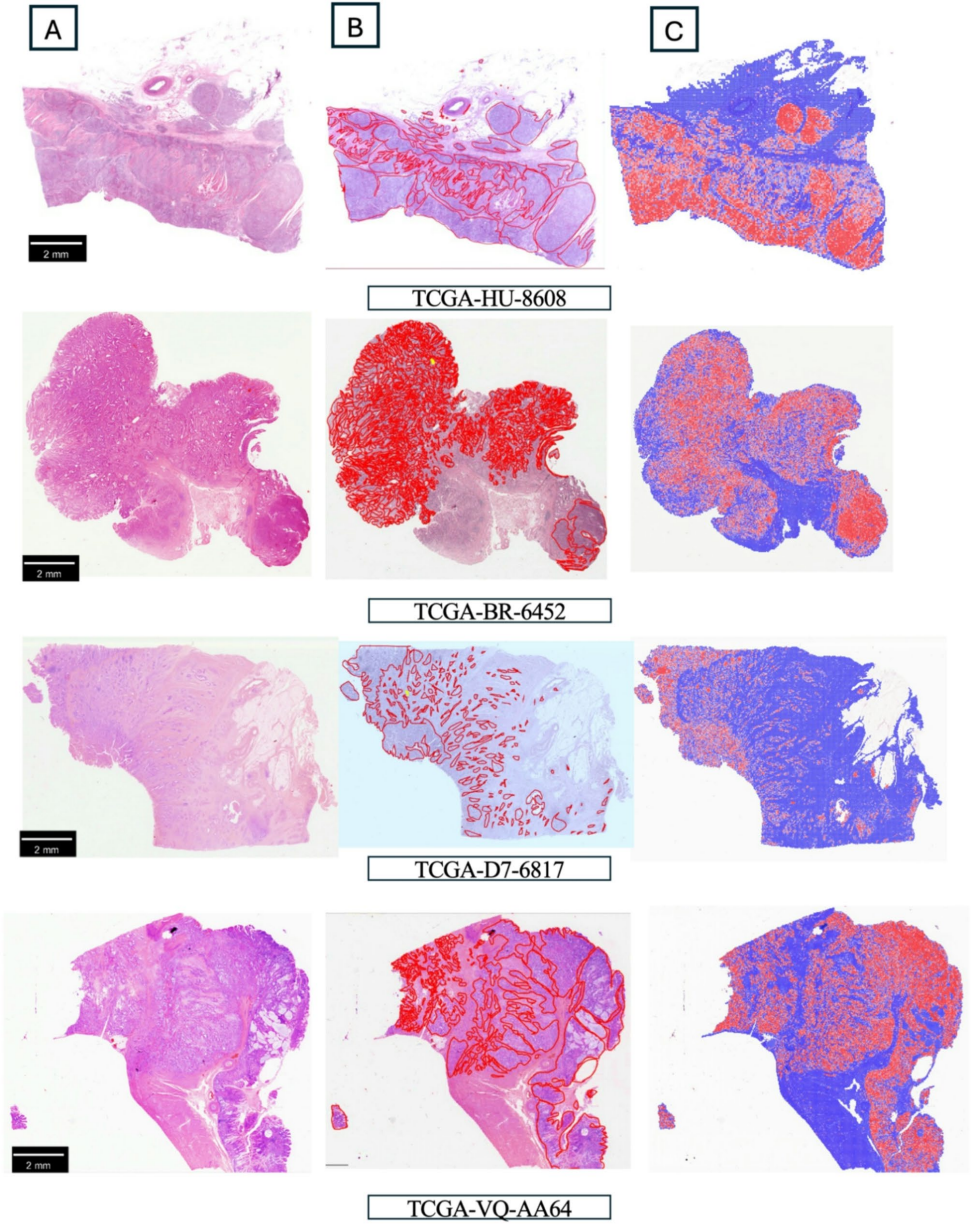
Visualization of the tumor detection model through heatmap visualization on a slide level. The model could predict the tumor area with high accuracy. **A.** The whole slide images from TCGA-STAD cohort. **B**. The annotation of tumor area by junior pathologist using QuPath version 5.1. **C**. Tumor area detected by tumor extraction model (red color)

The tumor extraction model demonstrated strong performance, achieving an AUROC of 0.99 across both the training dataset and an accuracy of 0.96 in dataset 1 and 0.97 in dataset 2. In the test set, the model achieved an AUROC of 0.91 and an accuracy of 0.84 in dataset 1, and an AUROC of 0.92 and an accuracy of 0.85 in dataset 2. On the external cohort, the tumor extraction model achieved an AUROC of 0.87 and accuracy of 0.87. The high specificity of 0.95 and high NPV of 0.90 indicate that the model is highly reliable at correctly identifying non-tumor regions. Details of the performance are shown in Table 2; Figs. 2 and 3, and supplementary Fig. 1.

**Table 2:** Tumor detection model performance

	Model	AUROC	Accuracy	F1 score	Specificity	Sensitivity	NPV	PPV
Training set	Dataset 1	0.99	0.96	0.96	0.97	0.96	0.96	0.97
	Dataset 2	0.99	0.97	0.97	0.98	0.96	0.97	0.97
Test set	Dataset 1	0.91	0.84	0.83	0.90	0.77	0.80	0.88
	Dataset 2	0.92	0.85	0.84	0.92	0.78	0.81	0.90
External test set	HiESD dataset	0.87	0.87	0.57	0.95	0.49	0.90	0.68

AUROC, area under the receiver operating characteristic curve; NPV, negative predictive value; PPV, positive predictive value

### Risk score prediction models

After extracting tumor tiles, we trained multiple risk score classification models using a dataset of 249 WSIs and evaluated their performances on an internal test set comprising 44 WSIs. The CNN model with four hidden-layers and CLAM models were trained using features derived from model dataset 2, whereas the DSMIL model was developed using the entire WSIs. A detailed comparison of the performances of these models is presented in Table 3.

**Table 3:** Comparison of different risk score prediction models

Model	5-fold cross-validation		Internal test set	
	AUROC	Accuracy	AUROC	Accuracy
CNN	0.5	0.51	0.56	0.57
CLAM	0.69	0.65	0.53	0.57
DSMIL	0.73	0.73	0.70	0.68

CNN, convolutional neural network; CLAM, clustering-constrained attention multiple-instance learning; DSMIL, dual-stream multiple-instance learning

**Fig. 4:**
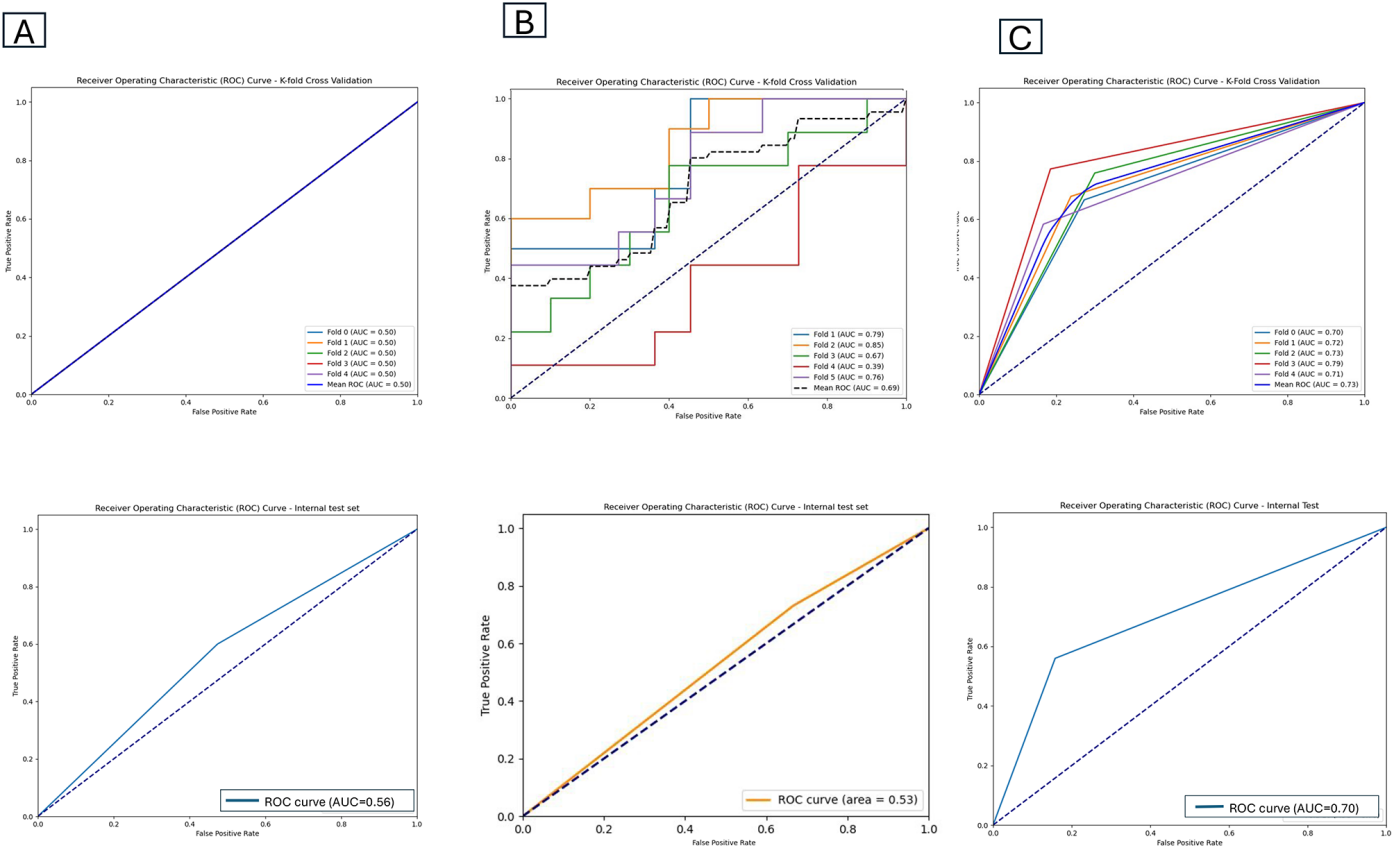
Performances of the risk score prediction models. **A-C** The receiver operating characteristic (ROC) curves and area under the curve (AUC) in the training set and internal test of convolutional neural network (CNN), clustering-constrained attention multiple-instance learning (CLAM), and dual-stream multiple-instance learning (DSMIL) models

Among the three models, DSMIL demonstrated the most robust performance in predicting prognoses and immunotherapy responses in GC, achieving an AUROC of 0.73 and an accuracy of 0.73 in the training set and an AUROC of 0.70 and accuracy of 0.68 in the test set, both higher than those of the CLAM and CNN models (Fig. 4). To statistically confirm this observation, we performed DeLong’s test to compare the AUROC of the model DSMIL against the CLAM model in the risk score prediction task. The analysis yielded a z-score of 2.10 and a *p*-value of 0.04. Since the *p*-value is less than 0.05, we concluded that the difference in is statistically significant, confirming the superior predictive capability of the DSMIL model.

### Visualizing predictive features: attention heatmap analysis

**Fig. 5:**
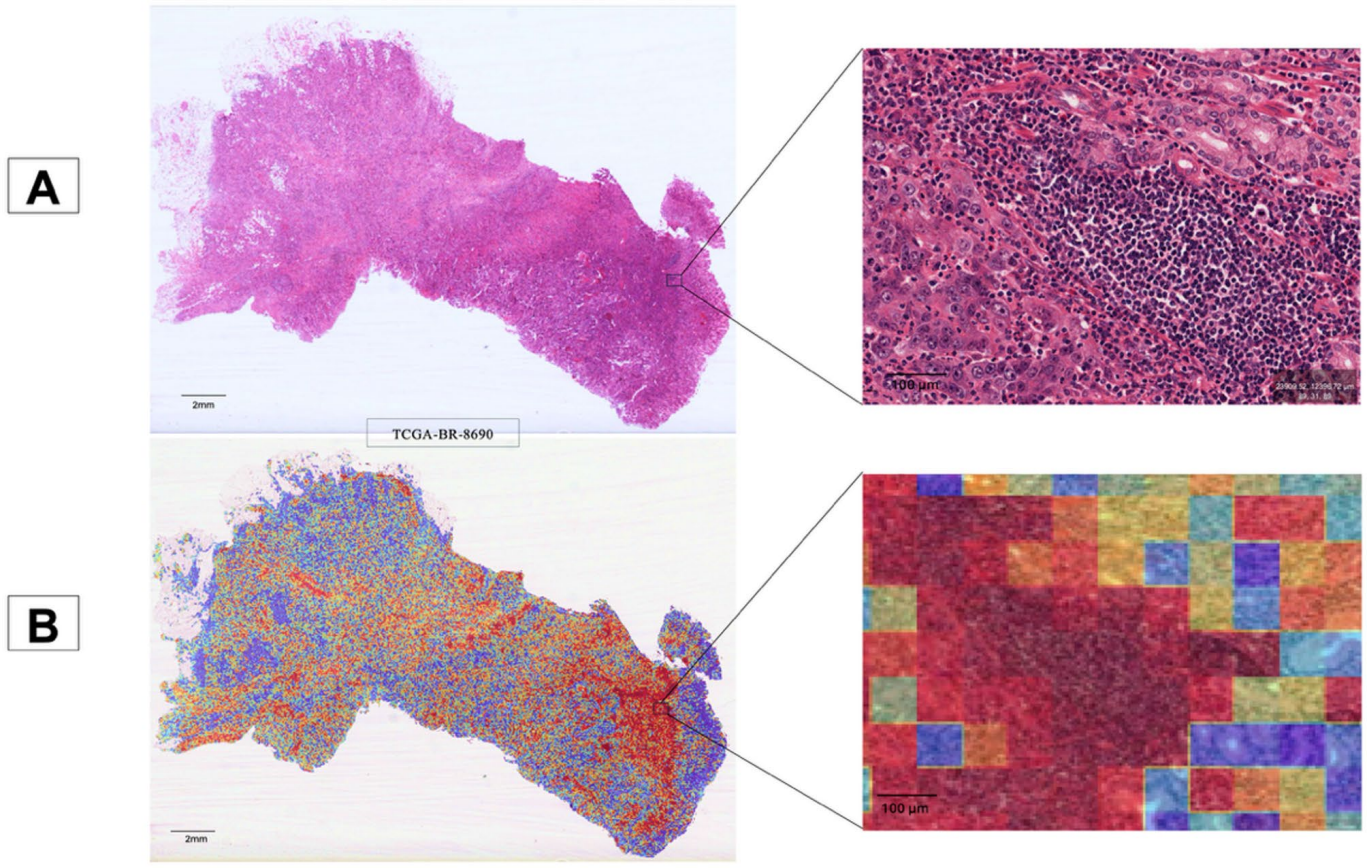
DSMIL attention heatmap for risk prediction **A** Whole slide image (WSI) illustrating the region of lymphocyte infiltration adjacent to the tumor. **B** The corresponding heatmap visualization where red intensity signifies the model’s highest confidence in predicting high risk within the lymphocyte infiltration area

To determine the specific morphological features contributing to the superior performance of the model in predicting the risk score, we conducted a feature attribution analysis by visualizing the attention heatmaps generated by the DSMIL architecture. The model’s ability to operate on WSI, rather than just isolated tumor regions, allowed for the implicit capture of complex TME components. The attention heatmaps consistently demonstrated that the predictive capacity of was heavily influenced by specific regions. Specifically, areas characterized by dense lymphocytic infiltration were assigned the highest attention scores by the DSMIL model (Fig. 5). This finding indicates that the model heavily relied on the visual cues of the patient’s immune and inflammatory response, particularly the infiltration of lymphocytes clustered around the tumor margin and within the stroma.

### Integration of deep features and clinicopathological data

We developed a multi-modal classification framework to investigate whether the integration of clinical factors could enhance the predictive performance achieved by image morphology alone. The results showed that the model combining SVM and DSMIL deep features alone achieved the highest performance, with an of 0.72 and an of 0.70. In comparison, the combined model SVM plus DSMIL plus clinical variables yielded a slightly lower performance, registering an of 0.70 and an of 0.68 (Table 4). Therefore, the model incorporating DSMIL deep features alone achieves the best overall predictive performance for the risk score prediction task, suggesting that the morphological features are the dominant predictive signal, and adding the available clinical variables did not improve discrimination in this specific configuration. A detailed description of the hyperparameter optimization process, including the full range of parameters tested for the SVM was showed in the Supplementary Tables 1 and 2 to ensure complete transparency and reproducibility of our methodology.

**Table 4:** Predictive performance of SVM models using deep features and clinicopathological variables

Method	AUROC	Accuracy	Sensitivity	Specificity	F1 score
SVM + clinical variable	0.61	0.57	0.68	0.42	0.64
SVM + DSMIL	0.72	0.70	0.76	0.63	0.75
SVM + DSMIL + clinical variable	0.70	0.68	0.76	0.58	0.73

## Discussions

GC remains a malignancy with a poor prognosis. Compared to conventional therapies, immunotherapy has demonstrated improved outcomes for patients with advanced GC. While ICIs have emerged as promising therapeutic options, only a small subset of patients derive significant benefits [26]. Predicting immunotherapeutic responses remains a major challenge due to tumor heterogeneity and limitations of conventional biomarkers, such as PD-L1 expression, TMB, and MSI [27]. Our previous study successfully identified a novel biomarker that integrates hypoxia and immune status, enabling predictions of both prognoses and immunotherapeutic responses in GC patients. We first developed a gene signature and calculated a risk score based on transcriptomic expression levels. Patients classified in the low-risk group exhibited better prognoses and greater responsiveness to immunotherapy. Building upon this foundation, in this study, we developed DL models capable of directly predicting prognoses and immunotherapeutic responses from histopathological images. To our knowledge, this is the first study to construct a DL model for predicting a novel biomarker that combines both hypoxia and immune status from pathological images.

There are many approaches for predicting responses to immunotherapy treatment for GC patients. In our research, we developed predictive models aimed at achieving high performance in predicting immunotherapeutic responses, utilizing patient risk scores. Results indicated that the DSMIL model outperformed the others, achieving the highest performance with an AUROC of 0.7 and an accuracy of 0.68 in the test set. Compared to study by Wei et al. [12], who developed DL models for predicting immunotherapeutic responses based on molecular features like the Epstein-Barr virus status, MSI, TMB, and PD-L1 expression, our results showed a clear contrast. Wei et al. reported AUROC values of 0.94 for EBV, 0.89 for MSI, 0.94 for TMB, and 0.86 for PD-L1 expression. Additionally, Liu et al. [13] created a DL model to predict the response to first-line PD-1 inhibitor combined chemotherapy for advanced-stage GC, achieving an impressive AUROC of 0.92. In contrast, our results were more favorable than those of Sun et al. [28], who constructed a DL model to predict non-apoptotic regulated cell death-related gene signatures in GC patients, with their model attaining an AUROC of 0.63. While these conventional markers have been extensively studied and validated in the context of immunotherapeutic responses, our approach introduces a novel prognostic and predictive biomarker that integrates both hypoxia and immune status. This innovative method shows great potential for clinical applicability, providing an improved framework for predicting immunotherapy outcomes in GC patients.

The TME has a crucial role in prognoses and predictions for immunotherapeutic responses for cancer patients [14, 29]. Pathological images typically include both tumor regions and surrounding normal tissues. However, in digital pathology, no studies have systematically evaluated how the selection of specific pathological regions in the preprocessing step affects model performance. For instance, to predict the prognosis of GC patients, Huang et al. [30] developed a GastroMIL model using WSIs, whereas Liu et al. [13] segmented regions of interest before inputting them into a model to predict treatment responses. In lung cancer, Park et al. [31] extracted features from tumor region segmentation models to predict gene mutations, while Ren et al. [32] extracted features from broader tissue regions before developing their model. In our study, we aimed to determine whether features extracted from the tumor region alone or from the entire tissue area of WSIs would impact the model performance. To address this, we tested different modeling approaches. First, we developed a tumor-detection model to identify tumor-specific tiles using two independent datasets. This model demonstrated high performance, achieving an AUROC of 0.92 and an accuracy of 0.85 on the test set. Furthermore, its robustness was confirmed through external validation on the HiESD dataset, where it achieved an AUROC of 0.87 and an accuracy of 0.87. Our findings aligned with those of Wei et al. [12], whose tumor extraction model achieved an AUROC of 0.94 on TCGA test set and 0.91 on an external dataset. While Wei et al. utilized a large dataset of 245,196 tiles from the GasHisDB, our study focused on a smaller subset, annotating a total of 18,000 tiles. This more-efficient annotation process was facilitated by concentrating on a carefully chosen subset of tiles, allowing us to obtain valuable results within a shorter time frame. Next, we applied the CLAM model to predict risk scores using features derived from the tumor extraction model, which resulted in an AUROC of 0.69 and an accuracy of 0.65. Additionally, we implemented the DSMIL model, which utilized WSIs for prediction. We found that the DSMIL model outperformed the tumor-based approach, achieving an AUROC of 0.73 for the training set and 0.70 for the internal test set. These findings suggested that incorporating the entire tissue area, rather than restricting analyses to tumor regions alone, improved the model performance.

The superior performance of the DSMIL model strongly suggested that the model was effectively integrating crucial information residing outside the primary tumor boundary. To mechanistically confirm this, we conducted feature attribution analysis by visualizing the attention heatmaps generated by the DSMIL architecture. The heatmaps consistently demonstrated that the regions assigned the highest attention scores by the model were those characterized by dense lymphocytic infiltration, rather than merely the solid tumor bulk, confirming that DSMIL is capable of translating subtle morphological manifestations of the patient’s immune status and TME into a robust prognostic prediction. Our findings are consistent with several studies suggesting that TILs serve a significant function in predicting the efficacy of cellular immunotherapy [33, 34].

Clinical factors such as age, gender, and pathological stage play a traditionally vital role in patient prognosis assessment and treatment stratification [35]. Our objective in developing a multi-modal classification framework was to investigate whether the integration of these established clinical factors could enhance the predictive capability achieved solely by image morphology. The results of this comparative analysis provided a compelling insight into the informational hierarchy between the two data modalities for risk score prediction. Specifically, the model utilizing DSMIL deep features alone demonstrated the optimal performance, achieving the highest and. Intriguingly, the performance slightly decreased when the available clinicopathological variables were combined with the deep features. This suggested that the predictive signal contained within the deep morphological features is so dominant that the addition of the traditional clinical data, in this specific cohort and configuration, either introduced redundant information or, more likely, introduced non-discriminatory noise that diluted the predictive focus of the SVM classifier.

Despite some strong identification results, the study has some limitations. The dataset was solely derived from TCGA, which may have limited the generalizability of the models to other populations with different clinical or pathological characteristics, and we need to validate the external dataset to obtain a more-robust model. While the tumor detection model performed well, the risk score classification models exhibited moderate predictive performances, particularly in the CLAM model, which showed reduced accuracy for the internal test set. While DSMIL outperformed the CNN and CLAM models, the relatively low AUROC values suggest room for improvement. The variability in performance across different models indicates that further refinement is needed.

## Conclusion

This study highlights the potential of DL models for predicting immunotherapeutic responses for GC using WSIs based on hypoxia and immune status. Our results demonstrated that a DL-based risk score classification model can effectively stratify patients into high-risk and low-risk groups, correlating with survival outcomes and responses to ICIs. Our findings also indicate that integrating TME features, especially dense lymphocytic infiltration improved predictive accuracy, offering a promising approach for refining immunotherapy selection in GC.

## Supplementary Information

Below is the link to the electronic supplementary material.

Supplementary Material 1

## Data Availability

The data that support the findings of this study was downloaded from TCGA-STAD cohort https://portal.gdc.cancer.gov/projects/TCGA-STAD. The code employed for the analysis in this paper is available from the corresponding author upon reasonable request.

## Abbreviations

GC: Gastric cancer
ICIs: Immune checkpoint inhibitors
WSIs: Whole-slide images
AI: Artificial intelligence
PD-L1: Programmed death-ligand 1
TMB: Tumor mutational burden
MSI: Microsatellite instability
TME: Tumor microenvironment
DL: Deep learning
CNN: Convolutional neural network
CLAM: Clustering-constrained attention multiple-instance learning
DSMIL: Dual-stream multiple-instance learning
AUROC: Area under the ROC curve
SVM: Support Vector Machine
