# Supplementary material for: Translational deep learning models for risk stratification to predict prognosis and immunotherapy response in gastric cancer using digital pathology
Source: J Transl Med. 2025 Dec 24;23:1419. doi: 10.1186/s12967-025-07416-z (PMC12729749; doi:10.1186/s12967-025-07416-z)
Supplement: Supplementary file 1 — Supplementary Material 1 [file 12967_2025_7416_MOESM1_ESM.docx]

**Supplementary Materials**

**Table 1.** Optimal performance metrics and parameters of the support vector machine (SVM) classifier, utilizing deep learning features extracted from the deep multiple instance learning (DSMIL) framework

| C | gamma | kernel | Accuracy | AUC | F1 | Sensitivity | Specificity |
| --- | --- | --- | --- | --- | --- | --- | --- |
| 0.001 | 0.01 | sigmoid | 0.57 | 0.27 | 0.72 | 1 | 0 |
| 0.001 | 0.01 | rbf | 0.57 | 0.31 | 0.72 | 1 | 0 |
| 0.001 | 0.1 | sigmoid | 0.57 | 0.29 | 0.72 | 1 | 0 |
| 0.001 | 0.1 | rbf | 0.57 | 0.31 | 0.72 | 1 | 0 |
| 0.001 | 1 | sigmoid | 0.57 | 0.3 | 0.72 | 1 | 0 |
| 0.001 | 1 | rbf | 0.57 | 0.5 | 0.72 | 1 | 0 |
| 0.001 | 10 | sigmoid | 0.57 | 0.3 | 0.72 | 1 | 0 |
| 0.001 | 10 | rbf | 0.57 | 0.5 | 0.72 | 1 | 0 |
| 0.001 | 100 | sigmoid | 0.57 | 0.3 | 0.72 | 1 | 0 |
| 0.001 | 100 | rbf | 0.57 | 0.5 | 0.72 | 1 | 0 |
| 0.01 | 0.01 | sigmoid | 0.7 | 0.27 | 0.79 | 1 | 0.32 |
| 0.01 | 0.01 | rbf | 0.57 | 0.31 | 0.72 | 1 | 0 |
| 0.01 | 0.1 | sigmoid | 0.64 | 0.71 | 0.72 | 0.84 | 0.37 |
| 0.01 | 0.1 | rbf | 0.57 | 0.3 | 0.72 | 1 | 0 |
| 0.01 | 1 | sigmoid | 0.61 | 0.7 | 0.7 | 0.8 | 0.37 |
| 0.01 | 1 | rbf | 0.57 | 0.5 | 0.72 | 1 | 0 |
| 0.01 | 10 | sigmoid | 0.61 | 0.7 | 0.7 | 0.8 | 0.37 |
| 0.01 | 10 | rbf | 0.57 | 0.5 | 0.72 | 1 | 0 |
| 0.01 | 100 | sigmoid | 0.61 | 0.7 | 0.7 | 0.8 | 0.37 |
| 0.01 | 100 | rbf | 0.57 | 0.5 | 0.72 | 1 | 0 |
| 0.1 | 0.01 | sigmoid | 0.59 | 0.7 | 0.64 | 0.64 | 0.53 |
| 0.1 | 0.01 | rbf | 0.57 | 0.31 | 0.72 | 1 | 0 |
| 0.1 | 0.1 | sigmoid | 0.57 | 0.65 | 0.58 | 0.52 | 0.63 |
| 0.1 | 0.1 | rbf | 0.57 | 0.3 | 0.72 | 1 | 0 |
| 0.1 | 1 | sigmoid | 0.59 | 0.62 | 0.61 | 0.56 | 0.63 |
| 0.1 | 1 | rbf | 0.57 | 0.5 | 0.72 | 1 | 0 |
| 0.1 | 10 | sigmoid | 0.59 | 0.6 | 0.61 | 0.56 | 0.63 |
| 0.1 | 10 | rbf | 0.57 | 0.5 | 0.72 | 1 | 0 |
| 0.1 | 100 | sigmoid | 0.55 | 0.61 | 0.55 | 0.48 | 0.63 |
| 0.1 | 100 | rbf | 0.57 | 0.5 | 0.72 | 1 | 0 |
| 1 | 0.01 | sigmoid | 0.55 | 0.66 | 0.57 | 0.52 | 0.58 |
| 1 | 0.01 | rbf | 0.66 | 0.69 | 0.69 | 0.68 | 0.63 |
| 1 | 0.1 | sigmoid | 0.52 | 0.57 | 0.43 | 0.32 | 0.79 |
| 1 | 0.1 | rbf | 0.57 | 0.3 | 0.72 | 1 | 0 |
| 1 | 1 | sigmoid | 0.43 | 0.54 | 0.39 | 0.32 | 0.58 |
| 1 | 1 | rbf | 0.57 | 0.48 | 0.72 | 1 | 0 |
| 1 | 10 | sigmoid | 0.48 | 0.55 | 0.44 | 0.36 | 0.63 |
| 1 | 10 | rbf | 0.57 | 0.5 | 0.72 | 1 | 0 |
| 1 | 100 | sigmoid | 0.43 | 0.53 | 0.39 | 0.32 | 0.58 |
| 1 | 100 | rbf | 0.57 | 0.5 | 0.72 | 1 | 0 |
| 10 | 0.01 | sigmoid | 0.59 | 0.65 | 0.61 | 0.56 | 0.63 |
| 10 | 0.01 | rbf | 0.7 | 0.72 | 0.75 | 0.76 | 0.63 |
| 10 | 0.1 | sigmoid | 0.48 | 0.57 | 0.41 | 0.32 | 0.68 |
| 10 | 0.1 | rbf | 0.57 | 0.3 | 0.72 | 1 | 0 |
| 10 | 1 | sigmoid | 0.41 | 0.53 | 0.32 | 0.24 | 0.63 |
| 10 | 1 | rbf | 0.57 | 0.48 | 0.72 | 1 | 0 |
| 10 | 10 | sigmoid | 0.5 | 0.55 | 0.48 | 0.4 | 0.63 |
| 10 | 10 | rbf | 0.57 | 0.5 | 0.72 | 1 | 0 |
| 10 | 100 | sigmoid | 0.57 | 0.59 | 0.56 | 0.48 | 0.68 |
| 10 | 100 | rbf | 0.57 | 0.5 | 0.72 | 1 | 0 |
| 100 | 0.01 | sigmoid | 0.61 | 0.66 | 0.62 | 0.56 | 0.68 |
| 100 | 0.01 | rbf | 0.7 | 0.72 | 0.75 | 0.76 | 0.63 |
| 100 | 0.1 | sigmoid | 0.45 | 0.58 | 0.4 | 0.32 | 0.63 |
| 100 | 0.1 | rbf | 0.57 | 0.3 | 0.72 | 1 | 0 |
| 100 | 1 | sigmoid | 0.41 | 0.53 | 0.32 | 0.24 | 0.63 |
| 100 | 1 | rbf | 0.57 | 0.48 | 0.72 | 1 | 0 |
| 100 | 10 | sigmoid | 0.41 | 0.56 | 0.35 | 0.28 | 0.58 |
| 100 | 10 | rbf | 0.57 | 0.5 | 0.72 | 1 | 0 |
| 100 | 100 | sigmoid | 0.48 | 0.58 | 0.38 | 0.28 | 0.74 |
| 100 | 100 | rbf | 0.57 | 0.5 | 0.72 | 1 | 0 |

**Table 2.** Optimal performance metrics and parameters of the support vector machine (SVM) classifier, utilizing deep learning features extracted from the deep multiple instance learning (DSMIL) framework and clinical variables

| C | gamma | kernel | Accuracy | AUC | F1 | Sentivity | Specificity |
| --- | --- | --- | --- | --- | --- | --- | --- |
| 0.001 | 0.01 | sigmoid | 0.57 | 0.27 | 0.72 | 1 | 0 |
| 0.001 | 0.01 | rbf | 0.57 | 0.31 | 0.72 | 1 | 0 |
| 0.001 | 0.1 | sigmoid | 0.57 | 0.29 | 0.72 | 1 | 0 |
| 0.001 | 0.1 | rbf | 0.57 | 0.33 | 0.72 | 1 | 0 |
| 0.001 | 1 | sigmoid | 0.57 | 0.29 | 0.72 | 1 | 0 |
| 0.001 | 1 | rbf | 0.57 | 0.5 | 0.72 | 1 | 0 |
| 0.001 | 10 | sigmoid | 0.57 | 0.29 | 0.72 | 1 | 0 |
| 0.001 | 10 | rbf | 0.57 | 0.5 | 0.72 | 1 | 0 |
| 0.001 | 100 | sigmoid | 0.57 | 0.29 | 0.72 | 1 | 0 |
| 0.001 | 100 | rbf | 0.57 | 0.5 | 0.72 | 1 | 0 |
| 0.01 | 0.01 | sigmoid | 0.7 | 0.27 | 0.79 | 1 | 0.32 |
| 0.01 | 0.01 | rbf | 0.57 | 0.31 | 0.72 | 1 | 0 |
| 0.01 | 0.1 | sigmoid | 0.64 | 0.71 | 0.72 | 0.84 | 0.37 |
| 0.01 | 0.1 | rbf | 0.57 | 0.32 | 0.72 | 1 | 0 |
| 0.01 | 1 | sigmoid | 0.64 | 0.71 | 0.71 | 0.8 | 0.42 |
| 0.01 | 1 | rbf | 0.57 | 0.5 | 0.72 | 1 | 0 |
| 0.01 | 10 | sigmoid | 0.64 | 0.71 | 0.71 | 0.8 | 0.42 |
| 0.01 | 10 | rbf | 0.57 | 0.5 | 0.72 | 1 | 0 |
| 0.01 | 100 | sigmoid | 0.64 | 0.71 | 0.71 | 0.8 | 0.42 |
| 0.01 | 100 | rbf | 0.57 | 0.5 | 0.72 | 1 | 0 |
| 0.1 | 0.01 | sigmoid | 0.59 | 0.7 | 0.64 | 0.64 | 0.53 |
| 0.1 | 0.01 | rbf | 0.57 | 0.31 | 0.72 | 1 | 0 |
| 0.1 | 0.1 | sigmoid | 0.57 | 0.62 | 0.58 | 0.52 | 0.63 |
| 0.1 | 0.1 | rbf | 0.57 | 0.32 | 0.72 | 1 | 0 |
| 0.1 | 1 | sigmoid | 0.59 | 0.61 | 0.61 | 0.56 | 0.63 |
| 0.1 | 1 | rbf | 0.57 | 0.5 | 0.72 | 1 | 0 |
| 0.1 | 10 | sigmoid | 0.59 | 0.61 | 0.61 | 0.56 | 0.63 |
| 0.1 | 10 | rbf | 0.57 | 0.5 | 0.72 | 1 | 0 |
| 0.1 | 100 | sigmoid | 0.61 | 0.63 | 0.64 | 0.6 | 0.63 |
| 0.1 | 100 | rbf | 0.57 | 0.5 | 0.72 | 1 | 0 |
| 1 | 0.01 | sigmoid | 0.55 | 0.66 | 0.57 | 0.52 | 0.58 |
| 1 | 0.01 | rbf | 0.66 | 0.69 | 0.69 | 0.68 | 0.63 |
| 1 | 0.1 | sigmoid | 0.45 | 0.59 | 0.4 | 0.32 | 0.63 |
| 1 | 0.1 | rbf | 0.57 | 0.32 | 0.72 | 1 | 0 |
| 1 | 1 | sigmoid | 0.5 | 0.55 | 0.5 | 0.44 | 0.58 |
| 1 | 1 | rbf | 0.57 | 0.5 | 0.72 | 1 | 0 |
| 1 | 10 | sigmoid | 0.45 | 0.58 | 0.4 | 0.32 | 0.63 |
| 1 | 10 | rbf | 0.57 | 0.5 | 0.72 | 1 | 0 |
| 1 | 100 | sigmoid | 0.45 | 0.56 | 0.4 | 0.32 | 0.63 |
| 1 | 100 | rbf | 0.57 | 0.5 | 0.72 | 1 | 0 |
| 10 | 0.01 | sigmoid | 0.55 | 0.62 | 0.55 | 0.48 | 0.63 |
| 10 | 0.01 | rbf | 0.68 | 0.71 | 0.73 | 0.76 | 0.58 |
| 10 | 0.1 | sigmoid | 0.45 | 0.6 | 0.4 | 0.32 | 0.63 |
| 10 | 0.1 | rbf | 0.57 | 0.32 | 0.72 | 1 | 0 |
| 10 | 1 | sigmoid | 0.48 | 0.56 | 0.47 | 0.4 | 0.58 |
| 10 | 1 | rbf | 0.57 | 0.5 | 0.72 | 1 | 0 |
| 10 | 10 | sigmoid | 0.45 | 0.59 | 0.4 | 0.32 | 0.63 |
| 10 | 10 | rbf | 0.57 | 0.5 | 0.72 | 1 | 0 |
| 10 | 100 | sigmoid | 0.48 | 0.58 | 0.47 | 0.4 | 0.58 |
| 10 | 100 | rbf | 0.57 | 0.5 | 0.72 | 1 | 0 |
| 100 | 0.01 | sigmoid | 0.61 | 0.65 | 0.62 | 0.56 | 0.68 |
| 100 | 0.01 | rbf | 0.68 | 0.71 | 0.73 | 0.76 | 0.58 |
| 100 | 0.1 | sigmoid | 0.45 | 0.6 | 0.4 | 0.32 | 0.63 |
| 100 | 0.1 | rbf | 0.57 | 0.32 | 0.72 | 1 | 0 |
| 100 | 1 | sigmoid | 0.48 | 0.55 | 0.47 | 0.4 | 0.58 |
| 100 | 1 | rbf | 0.57 | 0.5 | 0.72 | 1 | 0 |
| 100 | 10 | sigmoid | 0.43 | 0.56 | 0.36 | 0.28 | 0.63 |
| 100 | 10 | rbf | 0.57 | 0.5 | 0.72 | 1 | 0 |
| 100 | 100 | sigmoid | 0.48 | 0.58 | 0.47 | 0.4 | 0.58 |
| 100 | 100 | rbf | 0.57 | 0.5 | 0.72 | 1 | 0 |


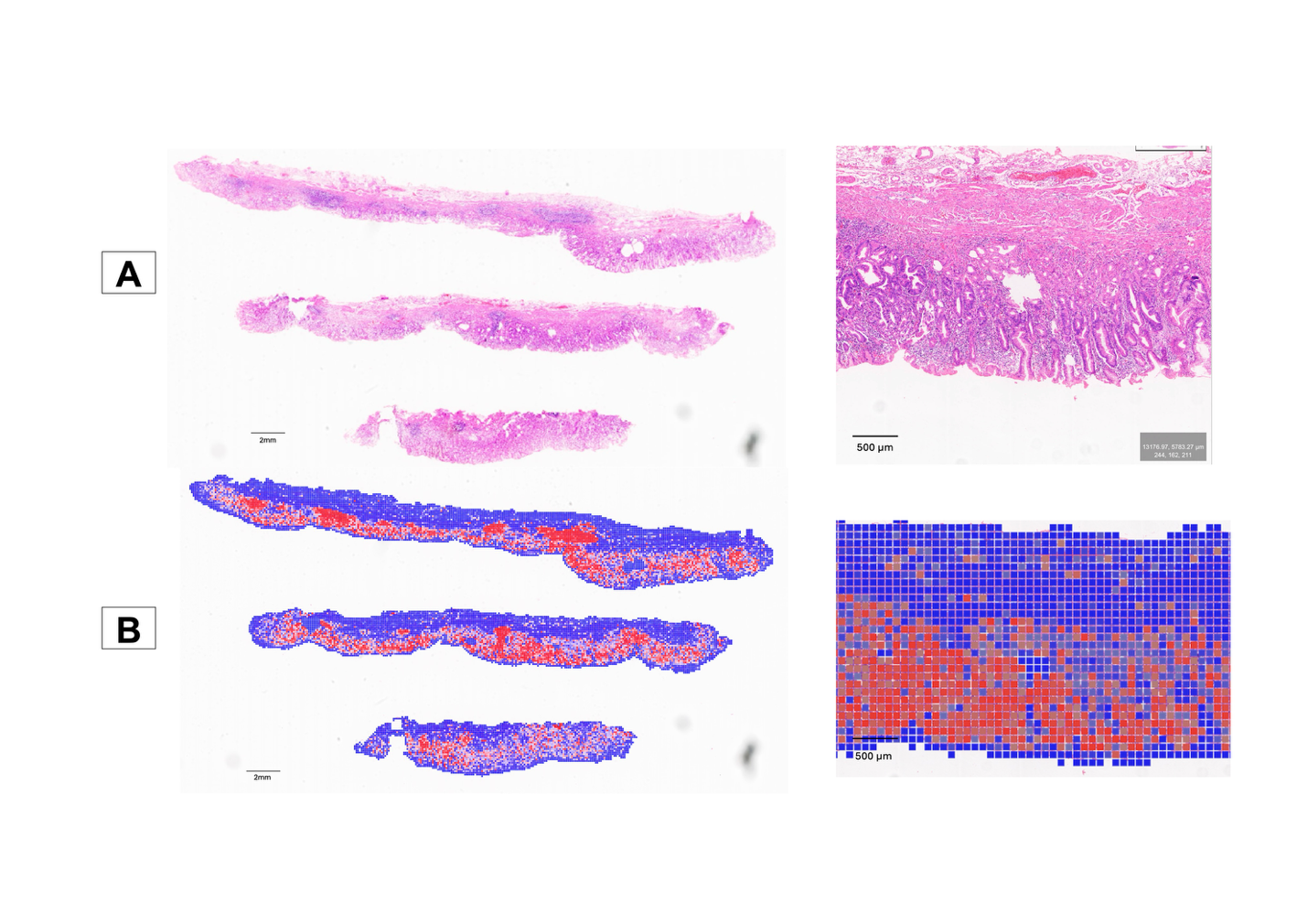


**Figure 1**. Heatmap visualization of tumor detection model on external validation HiESD dataset. **A.** H&E slide showing the tumor area. **B.** Model automatically detects the tumor area, visualized in red (Heatmap).
